# Eco-conscious potentiometric sensing: a multiwalled carbon nanotube-based platform for tulathromycin monitoring in livestock products

**DOI:** 10.1186/s13065-024-01255-7

**Published:** 2024-08-12

**Authors:** Omnia G. Hussein, Hany H. Monir, Hala E. Zaazaa, Maha M. Galal

**Affiliations:** Analytical Chemistry Department, Faculty of Pharmacy, Cairo University, Kasr El Aini Street, Cairo, 11562 Egypt

**Keywords:** Ion-selective electrodes, Tulathromycin, Multiwalled carbon nanotube, Potentiometric sensor, Whiteness, Livestock products, Veterinary drug residues, Food analysis

## Abstract

Tulathromycin (TUL) is a widely used veterinary antibiotic for treating bovine and porcine respiratory infections. Consuming animal-derived food contaminated with this medication may jeopardize human health. This work adopted the first portable potentiometric platform for direct TUL sensing in pharmaceutical and food products. The sensor employed a plasticized PVC membrane on a glassy carbon electrode doped with calix[6]arene and multi-walled carbon nanotubes (MWCNT) in a single solid contact layer for selective binding and signal stability. Characterization via scanning electron microscopy (SEM) and Fourier transform infrared spectroscopy (FTIR) confirmed the material’s integrity. The MWCNT-based sensor produced a stable Nernstian response (1.0 × 10^−7^ to 1.0 × 10^−3^ M) and a limit of detection (LOD) of 9.76 × 10^–8^ M with instantaneous response (8 ± 2 s). IUPAC validation revealed high selectivity for TUL against interfering ions, minimal drift (0.6 mV/h), and functionality over a broad pH range (2.0–7.0), allowing direct application to dosage form, spiked milk, and liver samples. Eco-Scale, AGREE, and Whiteness assessment proved the method's ecological sustainability, economic viability, and practical feasibility, surpassing traditional approaches.

**Graphical Abstract:**

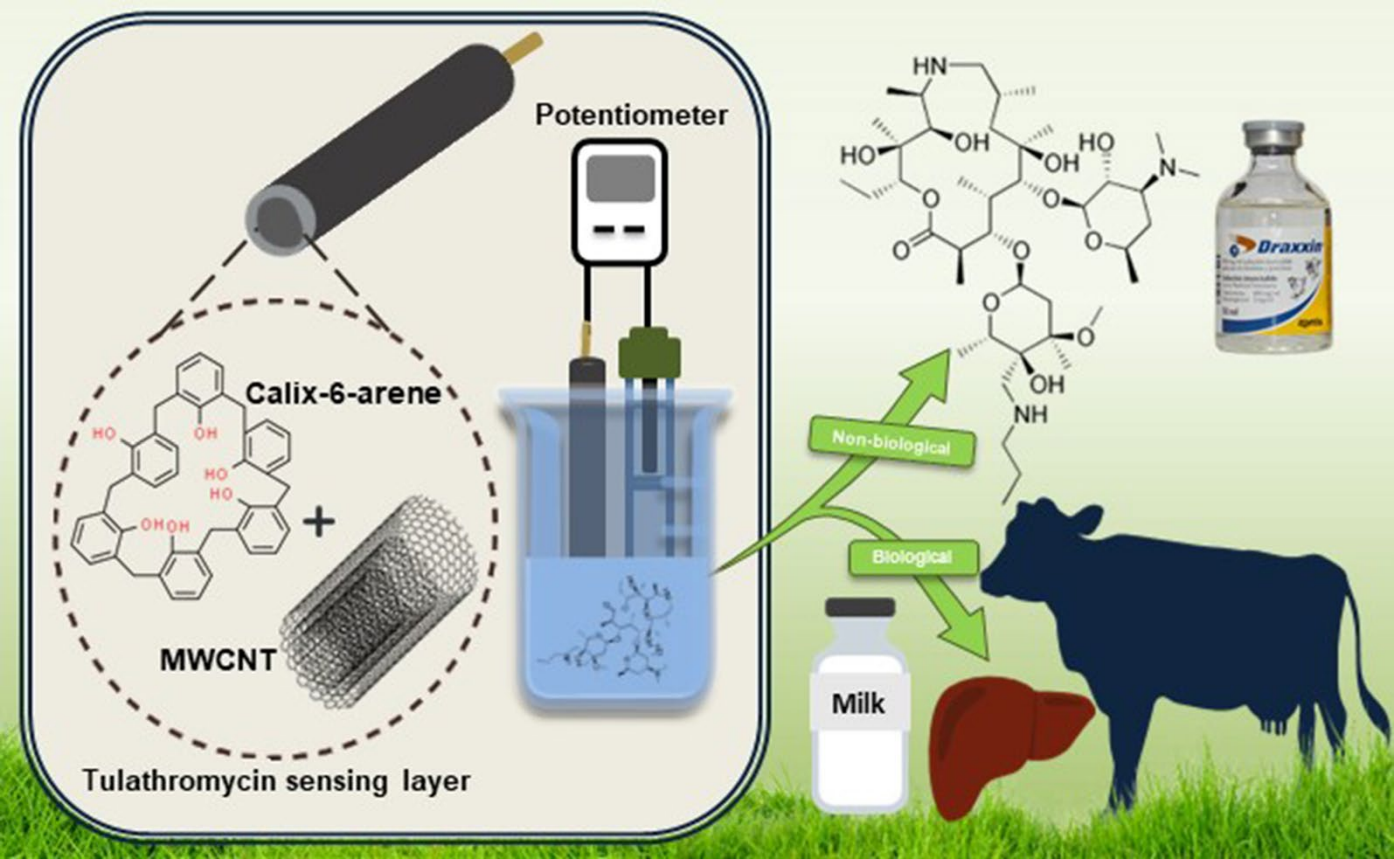

**Supplementary Information:**

The online version contains supplementary material available at 10.1186/s13065-024-01255-7.

## Introduction

The contamination of animal-derived foods with veterinary drugs, particularly antibiotics, has emerged as a major concern for human health. The excessive use of antibiotics and the lack of attention to antimicrobial withdrawal times can lead to the accumulation of drug remnants and their toxic metabolites in milk, meat, and other edible tissues [1]. One of the most critical consequences of these residues is the transmission of antibiotic-resistant bacteria, posing a profound threat to public health [2].

Tulathromycin (C_41_H_79_N_3_O_12_, TUL) (Fig. S-1) is a broadly utilized, long-lasting macrolide that belongs to the triamilide subclass. It is approved for treating respiratory infections in swine and cattle and is often administered to goats off-label [3, 4]. TUL is available as a sterile aqueous solution, characterized by expansive tissue distribution and a slow excretion half-life of 4–6 days, resulting in persistent drug concentration in the lungs [5]. Due to minimal metabolism, the unchanged drug predominates in edible tissues, urine, feces, and bile of treated animals. Notably, the liver accumulates the highest levels of TUL residues, making it a key matrix for investigation [6].

Given its persistence and capacity to accumulate in tissues (LogP 3.8), TUL poses formidable risks to human health and aquatic ecosystems [7, 8]. To ensure food safety, the European Union and other nations have established regulations governing TUL's maximum residue levels (MRL) [9]. Nevertheless, farmers continue to engage in inappropriate practices, such as administering antibiotics with incorrect doses or durations while overusing them for routine livestock management [10]. Consequently, acceptable thresholds of TUL are exceeded, causing adverse effects on consumers' well-being, including the potential development of allergies in hypersensitive people and the emergence of antibiotic-resistant pathogens [11]. Therefore, there is an urgent need for rapid and effective monitoring techniques for such compounds.

The analysis of TUL in biological matrices has heavily relied on chromatographic techniques, particularly liquid chromatography coupled with tandem mass spectrometry (LC–MS/MS) methods [4, 12–20]. Despite their high sensitivity and specificity, these methods suffer from significant limitations, entailing costly equipment, high energy consumption, the requirement for well-trained staff, extended analysis time, and arduous sample purification and preparation steps. On top of that, the need for expensive and environmentally detrimental organic solvents and the generation of toxic waste can restrict their practicality in routine analysis.

In contrast, electrochemical sensors, specifically solid-contact ion-selective electrodes (SC-ISEs), represent a cutting-edge analytical realm with miniaturized, simple, mobile, eco-friendly, and affordable devices [21]. These allow for speedy, accurate, and reproducible online monitoring of small volumes of samples with minimal pretreatment steps and thus have garnered attention across various scientific fields with prospective uses in medicine, environmental monitoring, and food analysis [22–24]. Based on the existing literature, no potentiometric sensor has been proposed for analyzing TUL. While an electrochemical voltammetric method was reported for determining TUL in pork samples [25], potentiometric sensing distinguishes itself by its passive operation that does not involve any external driving forces or redox reactions, low power, and hardly any sample consumption compared to the voltammetric approach [26, 27]. These attributes hold particular significance when the sample volumes are restricted; and the analyte concentration is low [28].

The optimization of the ion-selective membrane (ISM) begins with creating recognition sites capable of selectively binding the target ions in what is known as “host–guest chemistry” [29]. This study investigated two prominent classes of supramolecular ionophores: modified cyclodextrins and calixarenes, renowned for their capacity to form stable inclusion complexes with the analyte [30]. Incorporating nanomaterials has also revolutionized the development of electrochemical sensors, offering remarkable advancements in sensitivity, selectivity, and operational efficiency [31–34]. Carbon nanotubes, particularly multi-walled carbon nanotubes (MWCNT) and their nanohybrids, have served as the foundation for highly sophisticated sensing devices capable of detecting trace analytes across diverse domains such as medicine, food, agriculture, forensic and environmental research [35–39]. The distinctive structure of MWCNT imparts unique features to traditional carbonaceous electrodes, such as vast active surface area, exceptional electrical conductivity, rapid and efficient charge transfer with minimal resistance, signal amplification and stabilization, electrocatalytic properties, reduction of ISM overpotential, in addition to increased durability with chemical, light and thermal resistance [40]. This has led to a new generation of miniaturized sensors exhibiting high selectivity and lower detection limits. These sensors also eliminate the potential instability and irreproducibility associated with the formation of an aqueous layer, widely acknowledged as the major limitation of SC-ISEs [40–42]

For the first time, in this work, we aim to develop an eco-conscious, cost-effective, readily used solid-contact potentiometric sensor for accurate and sensitive determination of TUL in pharmaceutical formulations and animal food samples (bovine liver and milk). Central to our approach is exploring the synergistic potential of CX-6 and MWCNT within the sensor's membrane, aiming to optimize performance and enhance analytical capabilities.

## Experimental

### Instruments

An Adwa pH bench meter (AD1020 pH/mV/ISE/Temperature, Hungary), equipped with a glassy pH electrode (AD1131B, Hungary) and a double-junction Ag/AgCl reference electrode with an inner filling of 3.0 M KCl solution and outer filling of 10% KNO_3_ (Thermo Fisher Scientific, MA, USA), was set up for pH and potential measurements. A working glassy carbon electrode (GCE) (Ø 3 mm, CHI104) was bought from CH Instruments, Inc. (Texas, USA). WiseStir^®^ Magnetic Stirrer (DAIHAN Scientific, Seoul, Korea) was also utilized during experiments.

### Materials and reagents

Tulathromycin analytical standard was kindly provided by Pharma Swede (10th of Ramadan City, Egypt) with a certified purity of 97.35%. Draxxin^®^ injectable solution, stated to contain 100 mg of TUL per mL, was manufactured by Zoetis Belgium (Batch No. 548396). Deionized double-distilled water and pure analytical grade chemicals, solvents, and reagents were utilized. Polyvinyl chloride (PVC, high molecular weight), tetrakis(4-chlorophenyl)boron potassium ≥ 98% (KTCPB), 1-nitro-2-(n-octyloxy)benzene 98% (NPOE), dibutyl sebacate ≥ 97% (DBS), tritolyl phosphate 90% (TTP), dioctyl phthalate ≥ 99.5% (DOP), dibutyl phthalate 99% (DBP), calix[6]arene 97% (CX6), (2-hydroxypropyl)-B-cyclodextrin (average M_w_ ~ 1,460) (β-CD), calix[4]arene-25,26,27,28-tetrol 95% (CX4), 4-tert-butylcalix[8]arene ≥ 90% (tBu-CX8), tetrahydrofuran ≥ 99.9% (THF), multi-walled carbon nanotube powder (MWCNT) (≥ 98% carbon basis, O.D. × I.D. × L 10 nm ± 1 nm × 4.5 nm ± 0.5nm × 3- ~ 6 μm, TEM) was supplied by Sigma-Aldrich (Germany); glacial acetic acid 100% and sodium hydroxide pellets were provided by Merck (Germany), boric acid 85% from El-Nasr Chemicals Co., Egypt, and orthophosphoric acid (abt. 85% LR) from S.D.Fine-Chem Ltd., India.

### Solutions

A stock standard solution of TUL (1.0 × 10^–2^ M) was prepared by dissolving 80.6 mg of TUL in Britton-Robinson buffer (BRB) with pH (4.0 ± 0.2) into a 10-mL measuring flask. From this solution, precise aliquots were withdrawn to prepare a working series of dilutions (1.0 × 10^–9^–1.0 × 10^–3^ M) utilizing the same buffer. A stock MWCNT suspension (1.0 mg mL^−1^) was obtained by sonicating MWCNT in THF for at least 30 min. Prior to each use, the suspension was sonicated for 10 min to achieve equal dispersion. BRB buffer was made by adding 0.04 M of phosphoric acid and acetic acid to 0.04 M boric acid, followed by pH adjustment to 4.0 using 1.0 M NaOH solution.

### Fabrication of SC-ISE sensors

Two sensing membrane cocktails were prepared. Sensor 1 comprised 10.0 mg KTCBP, 10.0 mg of CX6, and 190.0 mg PVC mixed with 0.39 mL of NPOE plasticizer, all in 4.0 mL THF in a glass tube. For sensor 2, 0.36 mL of MWCNT suspension was introduced to 1.0 mL of the previously prepared cocktail and sonicated for 20 min to attain a homogenous suspension, following established reports [43, 44].

The GCEs were initially polished using 0.3 µm Al_2_O_3_ slurry, washed, sonicated in distilled water for 5 min to remove alumina traces, and then dried at room temperature. Fifteen µL of each sensing mixture was drop-casted on the surface of the polished GCEs and allowed to dry overnight. All sensors were preconditioned by soaking in 1.0 × 10^–4^ M TUL solution for 2 h before calibration.

### Potentiometric measurement

For the calibration of TUL sensors, each electrode, coupled with the silver chloride reference electrode, was placed into working standard solutions (1.0 × 10^–9^–1.0 × 10^–3^ M) and stirred to equilibrate. Upon reaching a steady response, the potentiometer's reading was taken within ± 1 mV. After each measurement, the membranes were rinsed with BRB buffer. The obtained potential (E, mV), which refers to the galvanic potential difference recorded between the ion-selective GCE electrode and the Ag/AgCl reference electrode, was graphed in relation to the logarithmic concentration values of TUL solutions to construct calibration curves.

The regression equation was derived from the linear part of the calibration curve, and the detection limit (LOD) was determined at the crossover point of extrapolated lines at the lower concentration segment of the curve.

The method’s accuracy was verified by determining five distinct concentrations of the TUL standard; the method's precision was validated by repeating the measurements of three distinct concentrations of the TUL standard three times in a single day to evaluate the repeatability and on three consecutive days to assess the method's intermediate precision.

### Sensors’ selectivity

We employed the separate solutions method (SSM) to examine the selectivity of the two suggested sensors for TUL with the occurrence of interfering ions [45]. The study investigated common ions and pollutants possible in targeted matrices, including K^+^, Na^+^, Ca^2+^, and Mg^2+^, as well as some compounds structurally related to TUL. The ability of sensing membranes to distinguish between the primary ion of interest and other ions with the same charge sign was calculated and expressed as the logarithm of the selectivity coefficient:

$${\mathbf{l}\mathbf{o}\mathbf{g}({\varvec{K}} }_{{\varvec{p}}{\varvec{r}}{\varvec{i}}{\varvec{m}}{\varvec{a}}{\varvec{r}}{\varvec{y}} {\varvec{i}}{\varvec{o}}{\varvec{n}}, {\varvec{i}}{\varvec{n}}{\varvec{t}}{\varvec{e}}{\varvec{r}}{\varvec{f}}{\varvec{e}}{\varvec{r}}{\varvec{e}}{\varvec{n}}{\varvec{t}}}^{{\varvec{p}}{\varvec{o}}{\varvec{t}}})=\frac{\left({E}_{I}-{E}_{TUL}\right){ Z}_{A}F}{2.303RT}$$

where *E*_*I*_ and *E*_*TUL*_ are the potential readings (mV) in 10^–3^ M solution of interferent species and primary ion (TUL), respectively, 2.303RT*/Z*_*A*_*F* corresponds to the slope of the suggested sensor (mV/decade).

### Effect of pH and the dynamic response time

The impact of pH changes within the range (2.0–10.0) on recorded potential values was examined by gradually introducing tiny portions of 1.0 M NaOH solution to 1.0 × 10^–3^ M and 1.0 × 10^–4^ M TUL solutions initially prepared at pH = 2.0 and measuring the electromotive force (emf) at each pH value.

To determine the practical response time, the duration needed to attain a stable potential reading (± 1 mV) after the change of TUL concentration was recorded.

### Potentiometric determination in pharmaceutical formulation

A quantity equivalent to 100 mg TUL (1.0 mL) was accurately transferred from Draxxin^®^ Injectable solution (100 mg. mL^−1^) into a 50-mL measuring flask and completed with BRB buffer (pH 4.0). This solution was then diluted by the same buffer to achieve final TUL concentrations of 4.96 × 10^–5^ M and 4.96 × 10^–4^ M. The optimized sensor was immersed in the prepared solutions, the emf measurements were recorded, and the regression equation was employed to find the respective concentration.

### Potentiometric determination in spiked milk and liver

Fresh liver and pasteurized milk samples were purchased from a local butcher and supermarket in El Sayeda Zeinab district of Cairo Governorate. One gram of chopped liver was accurately weighed, spiked with a known amount of TUL, and then homogenized in 25 mL BRB buffer (pH = 4.0). For milk samples, a volume of 2.5 mL was taken into 25 mL of BRB buffer without further treatment. The samples were spiked to achieve concentrations ranging from 1.0 × 10^–4^ to 1.0 × 10^–6^ M, below and above TUL specified MRL. Direct potentiometric measurements were carried out using the optimized sensor as previously described while swiftly alternating between spiked samples and distilled water during the experiment to prevent biological matrix components from clinging to the sensing surface. Five replicates of each concentration were analyzed, and an un-spiked (blank) sample served as a control.

Calibration graphs were generated by establishing a correlation between the recorded emf values and the logarithm of molar concentrations of TUL. The potential values for each sample were noted, and the found concentrations of TUL were computed using the respective regression equation.

## Results and discussion

In response to the rising demand for feasible analytical methods to detect antibiotics in various food products, we focused on developing a reliable and cost-effective sensor for the targeted analysis of TUL macrolide in different matrices while considering environmental safety and sustainability standards.

Initially, we attempted to create a sensing membrane cocktail with optimal binding affinity for TUL by screening various ion exchangers, plasticizers, and ionophores. We then exploited the excellent electrochemical conductivity of MWCNT to stabilize the response and improve sensitivity, ensuring compliance with TUL regulations in targeted tissues. The performance metrics of the developed sensors were evaluated based on IUPAC guidelines. Finally, the optimized sensor was employed to analyze TUL in dosage form, spiked milk, and liver samples.

### Membrane composition and effect of plasticizers

TUL predominantly behaves as a trivalent cation under acidic conditions, Fig. S-1 [46, 47]; therefore, the cationic exchanger KTCBP was paired with an NPOE plasticizer as the best combination for good baseline potentiometric response.

We started by examining the influence of several plasticizers, namely NPOE, DOP, DBP, DBS, and TTP, on the performance of electrodes. The calibration charts revealed that the use of NPOE achieved the best linear response and the highest sensitivity for the membrane, Fig. S-2 NPOE, DBS, and DBP showed better slopes (20.1, 20.4, and 21.4 mV/decade) than TTP and DOP (24.0 and 31.1 mV/decade). Meanwhile, NPOE demonstrated the best correlation coefficient (0.9998) and lowest LOD (2.38 × 10^–7^ M) compared to other plasticizers. These results can be attributed to variations in polarity/dielectric constants and functional groups of different plasticizers [48].

### Ionophore selection

Ionophores are crucial components of ISEs, serving as sites for chemical recognition. A strong binding affinity between the ionophore and target ion forms an inclusion complex with reduced solvation free energy, facilitating its transfer from the hydrophilic sample solution across the hydrophobic membrane [49]. This host-and-guest intermolecular interaction promotes sensor selectivity. Additionally, the high lipophilicity of the formed complex reduces the leaching of target ions into the aqueous phase, extending the sensor lifespan [50].

Cyclodextrins and calixarenes are widely recognized ionophores in ISE research. Calixarenes are macrocyclic molecules with aromatic basket-shaped cavities. These cavities have the capacity to establish unique interactions with target ions, such as cation-π interactions, π—π interactions, van der Waals interactions, and hydrogen bonding. The macrocyclic conformation and cavity size significantly impact host–guest binding and sensing membrane selectivity [50, 51].

**Fig. 1:**
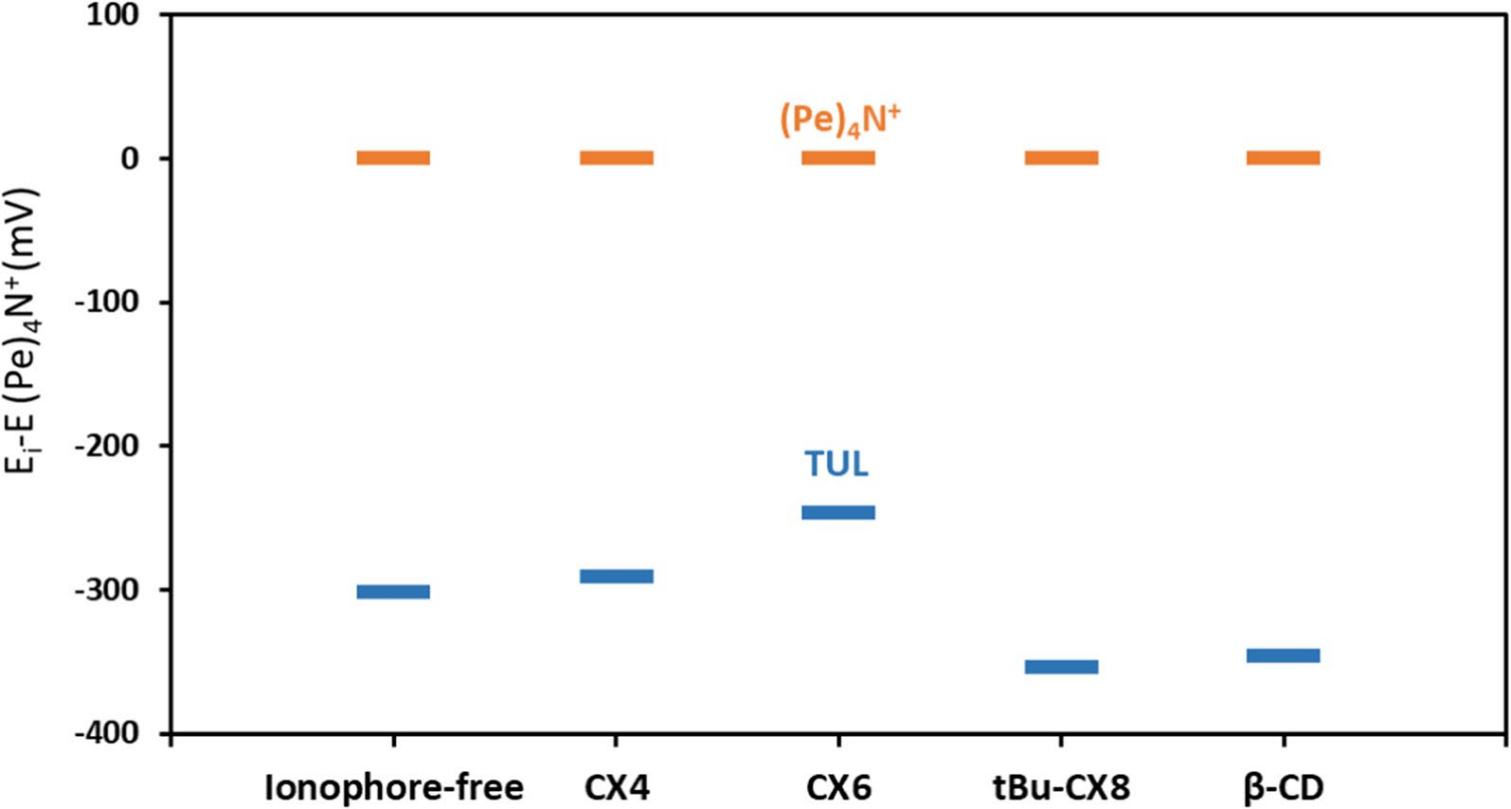
The potentiometric response of ISEs containing various ionophores in 1.0 mM TUL solution after subtracting each ISE's reading to 1.0 mM (Pe)_4_N^+^ solution

We investigated the recognition capacity of various calixarene derivatives for TUL, namely CX4, CX6, and tBu-CX8, in addition to β-CD, compared to an ionophore-free membrane. The sensors’ readings for TUL were measured against a reference ion, Tetra-n-pentylammonium bromide ((Pe)_4_N^+^)Br^−^ [49]. (Pe)_4_N^+^ is a bulky lipophilic ion, incapable of fitting inside the calixarene molecular cavity, thus helping identify the calixarene molecule with the highest TUL affinity. The efficiency of TUL-ionophore binding is maximized when the emf difference from (Pe)_4_N^+^ is minimized. As illustrated in Fig. 1. CX6-doped ISM achieved the highest binding and the lowest emf differential (247 mV) as opposed to other ionophores (CX4 291 mV, tBu-CX8 354 mV, and β-CD 346 mV) and the ionophore-free sensor (302 mV). Thus, CX6 was assigned as the ionophore of choice in later experiments. Fig. S-3 shows a schematic for the interaction between the TUL ion and the active sites in the membrane, predicting the occurrence of π—π interactions and H-bonding between the carbon tail of the oxane ring in TUL and the carbon and oxygen centers in CX-6.

### MWCNT incorporation and characterization

Incorporating MWCNT as a transducing layer in ISMs has been suggested to improve stability, reduce signal drift, and enhance membrane sensitivity [43]. We adopted a feasible approach, where MWCNT was dispersed into the NPOE plasticized membrane via ultrasonic vibrations without surfactants [44]. We doped the sensing membranes with different ratios of MWCNT (0.21, 0.36, 0.42 mg.mL^−1^) to investigate the effect on potential stability and sensitivity. The most promising outcome in terms of slope and linearity range was achieved at a concentration of 0.36 mg.mL^−1^, Fig. S-4.

**Fig. 2:**
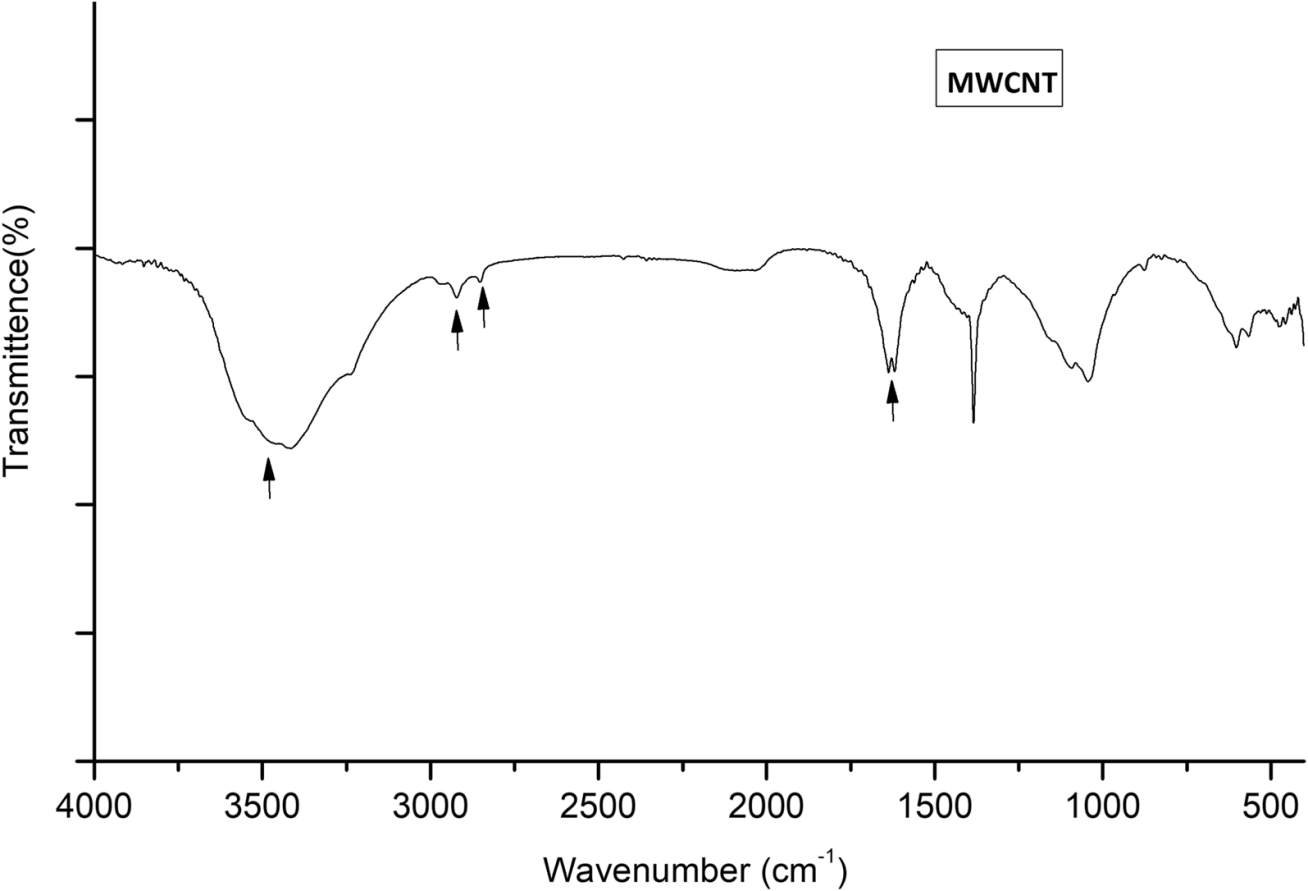
FT-IR spectrum of pristine MWCNT using KBr pellets in the 4000–400 cm^−1^ range

The structural features of MWCNT powder were examined using FT-IR spectroscopy following its placement onto potassium bromide (KBr) discs. Figure 2 reveals a characteristic peak at 1636 cm^−1^ that indicates the occurrence of the aromatic C=C stretching vibration in the graphite arrangement of the MWCNT framework. A five/seven-membered presence at the junction or closure of the MWCNT is responsible for this phenomenon [52]. Bands of symmetric/asymmetric –CH_2_ methylene stretching are observed at 2922 and 2852 cm^−1^ [53]. The spectrum shows strong bands at 3400 cm^−1^, corresponding to the surface–OH stretching vibrations caused by either atmospheric moisture adsorption or oxidation during the purification of nanoparticles [54].

**Fig. 3:**
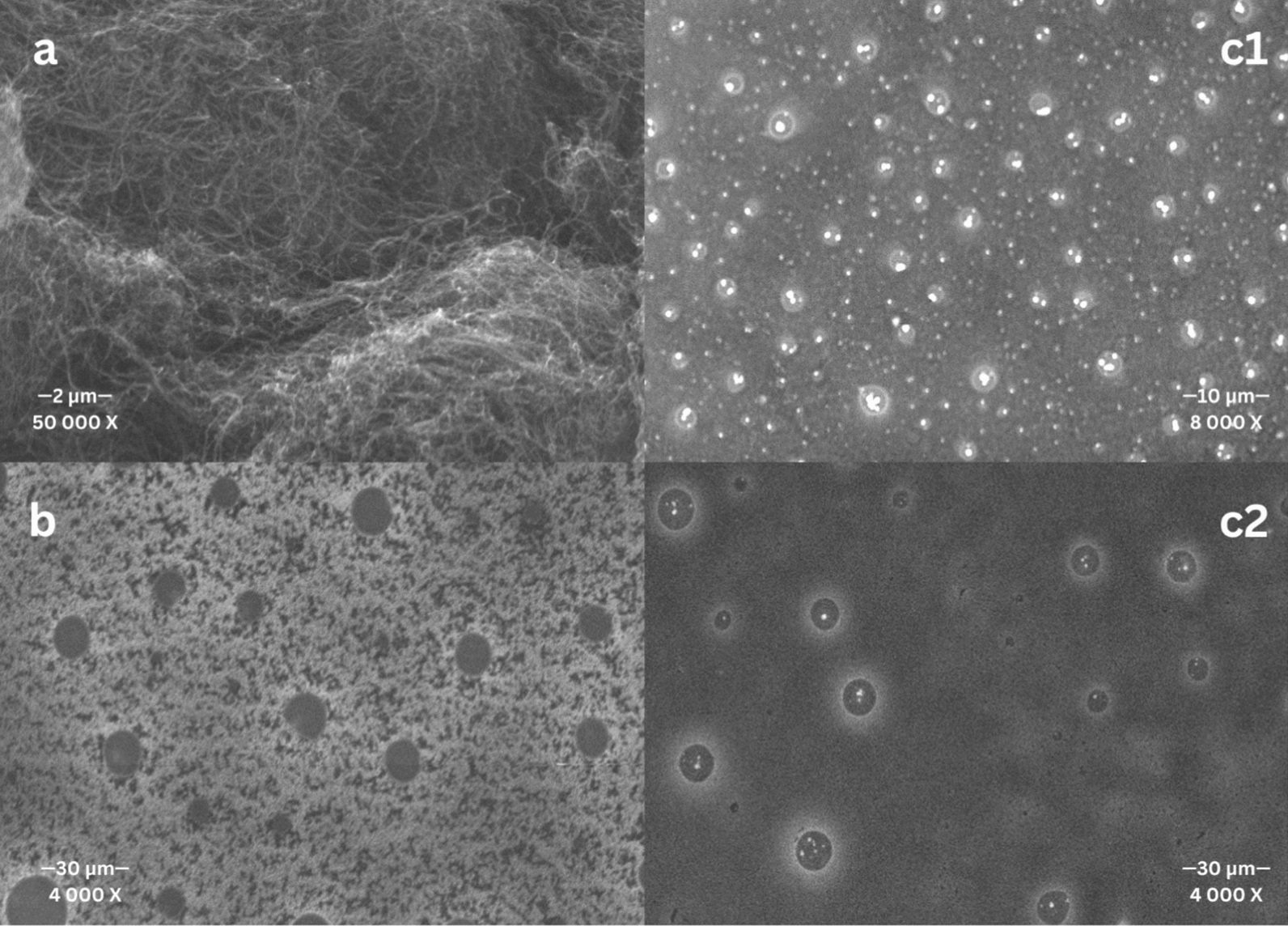
Surface SEM images of **a** raw MWCNT, **b** unmodified ISM, and **c1,2.** MWCNT/ISM

Scanning electron microscopy (SEM) (Quanta FEG 250) was employed to analyze the surface morphology of MWCNT and the prepared membranes. Figure 3a shows networks of intertwined nanofibers of raw MWCNT at high magnification. Before doping with MWCNT, the polymeric nature of the unaltered membrane with an even distribution of pores across the surface is displayed in Fig. 3b. The difference after MWCNT inclusion is evident in Fig. 3c2 (30 kV), where white particles encapsulated within the membrane pores appear brighter due to differences in conductivity. More surface details of the MWCNT/ISM are observed in Fig. 3c1 (10 kV), revealing the uniform dispersion of nanoparticles within the membrane matrix. This modification in the geometrical surface of the electrode might create an expanded electrochemical active area for TUL sensing.

### Performance characteristics and validation parameters of the developed sensors

**Fig. 4:**
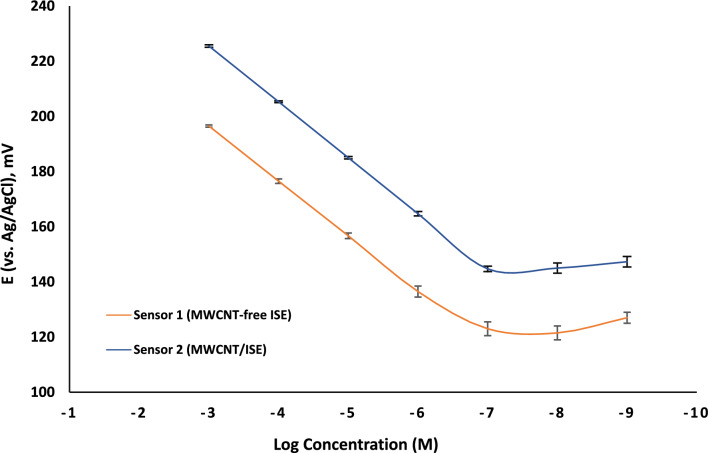
The plot of change in potential (mV) as a function of logarithmic molar concentrations of TUL employing sensors 1 and 2

We investigated the analytical capabilities of the two designed sensors, MWCNT-free ISM (sensor 1) versus MWCNT/ISM (sensor 2), by graphing the emf measurements with respect to the logarithmic concentrations of TUL solutions in Fig. 4. Then, the calibration parameters were extracted and compared in Table 1 according to IUPAC recommendations [55]. The sensors’ analytical characteristics demonstrated a significant performance enhancement following the incorporation of MWCNT. Results exhibited a wider linearity range and lower detection limit for sensor 2 (1.0 × 10^–3^–1.0 × 10^–7^ M, LOD 9.76 × 10^–8^ M) over sensor 1 (1.0 × 10^–3^–1.0 × 10^–6^ M, LOD 1.73 × 10^–7^ M). This suggests a ten-fold enhancement in signal quantification upon the addition of MWCNT. Lower detection limits may be linked to the role of MWCNT in hindering the formation of the aqueous layer that can accumulate TUL ions and cause leaching into more diluted concentrations during measurement.

**Table 1:** The electrochemical validation parameters of the designed GCE sensors

Parameters	Sensor 1 (MWCNT-free ISM)	Sensor 2 (MWCNT/ISM)
Slope (mV/decade)^a^	19.98	20.22
Intercept (mV)	256.46	286.14
Correlation Coefficient (r)	1	1
Linearity Range (M)	1.0 × 10^–6^–1.0 × 10^–3^	1.0 × 10^–7^–1.0 × 10^–3^
Working pH Range	2–7	2–7
Response Time (Sec.)	18	8
Lifetime (Weeks)	3	7
Accuracy (Mean ± RSD%)^b^	99.84 ± 0.700	100.23 ± 0.576
Repeatability^c^ (%)	1.037	1.011
Intermediate Precision^c^ (%)	1.619	1.486
Robustness^d^ (%)	1.264	0.986
LOQ (M)	1.0 × 10^–6^	1.0 × 10^–7^
LOD^e^ (M)	1.73 × 10^–7^	9.76 × 10^–8^

^a^Average of three measurements

^b^Mean ± RSD% of recoveries for five concentrations of TUL in triplicates

^c^ RSD% values for three concentration levels (1.0 × 10^–4^, 1.0 × 10^–5^, 1.0 × 10^–6^ M) of TUL, each repeated three times intraday for repeatability and interday for three consecutive days for intermediate precision

^d^RSD% values for previous concentrations under minor pH variations

^e^LOD (detection limit) was determined by intercepting the extrapolated lines of non-responsive and the Nernstian sections in the potential profile

The calculated percentage mean recoveries and relative standard deviations are within the acceptable limits, verifying the sensors’ accuracy, repeatability, and precision, with slightly favorable values in the case of sensor 2 (MWCNT/ISM).

The MWCNT/ISM sensor also displayed superior features, including greater potential stability, lower drift, faster response time, and longer shelf life, as discussed in later sections. All these characteristics would be crucial for the analytical applications of a novel sensor, particularly in biological matrices.

### Dynamic response times and sensors’ lifetimes

The dynamic response time curve reflects both the time the sensor takes to attain equilibrium and the stability of the response. The potential-time curve for MWCNT/ISM in Fig. S-5 displays an immediate response with satisfying potential stability to alterations in TUL concentration within 3 s at high concentration levels. At lower concentration levels, the response time increased to 10 s to stabilize within 1 mV of the steady-state potential. This results in an average response time of (8 ± 2) seconds for sensor 2, in contrast to (18 ± 2) for sensor 1, indicating that the inclusion of MWCNT improved both reaction time and signal stability. Moreover, this instantaneous response emphasizes the efficacy of ISEs over conventional chromatographic techniques, especially for applications requiring rapid analysis of numerous samples.

Repeated calibrations consistently yielded a reproducible slope of ± 1 mV/decade over a 3-week period for sensor 1, with frequent incidents of membrane cracking and peeling, in contrast to sensor 2, which maintained stability for seven weeks (Table 1). This proves that the C − C bonds forming nanotubes in MWCNT impart exceptional strength, flexibility, and resistance to membrane rupture. It underscores the role of MWCNT in enhancing the sensor's durability and broadening its potential applications.

### Effect of soaking time

To explore the impact of preconditioning time on the measured response, the sensors were immersed in 1.0 × 10^–4^ M of TUL for various periods ranging from 1 to 48 h. Interestingly, soaking the sensor for 2 h up to 12 h showed comparable results in contrast to soaking for a longer time, which resulted in an unfavorable decline in slope and detection limit. The observed decline may be attributed to the leakage of electroactive molecules into the soaking solution. Therefore, a duration of 2 h was set to be the optimal soaking time.

### Effect of pH

**Fig. 5:**
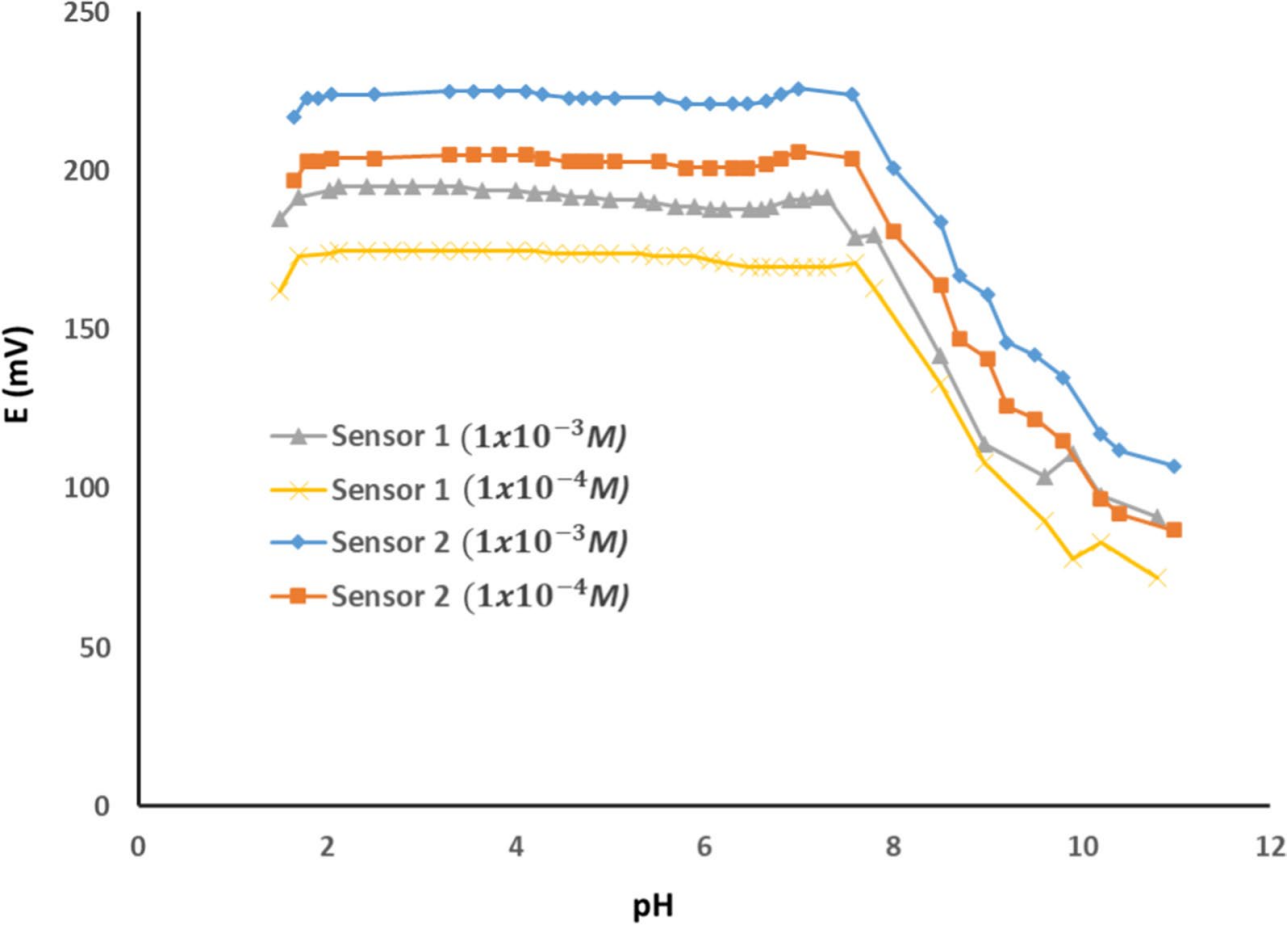
Potential pH profile of the proposed sensor 1 (MWCNT-free sensor) and sensor 2 (MWCNT/ISE) using 1.0 × 10^–3^ M and 1.0 × 10^–4^ M TUL solutions

TUL pH-potential profile was evaluated to establish the optimal operating parameters for the sensors under investigation. A steady electric potential prevails over a wide pH range of 2.0 to 7.0 for both sensors, as shown in Fig. 5. TUL possesses three primary amine groups that undergo ionization in acidic conditions, as indicated by their dissociation constants (pK_a_ of 8.59, 9.62, and 9.90) [47]. This contrasts with an alkaline pH, where the three amines become less positively charged, explaining the gradual decline in the measured potential with the subsequent increase in pH above 7.5. Therefore, pH 4.0 was employed during measurements to ensure the complete ionization of TUL.

### Sensors’ selectivity

We assessed the selectivity of both ISE sensors against potentially interfering ions in biological matrices of interest by applying the separate solution method (SSM) [45]. The calculated selectivity coefficients are expressed as (${{\varvec{l}}{\varvec{o}}{\varvec{g}} {\varvec{K}} }_{{\varvec{T}}{\varvec{U}}{\varvec{L}}. {\varvec{I}}}^{{\varvec{p}}{\varvec{o}}{\varvec{t}}}$), and are presented in Table 2. The values range from -2.99 to -3.83, indicating a high level of selectivity towards TUL without significant interference from inorganic species such as Na^+^, K^+^, Ca^+^, and Mg^2+^. The high selectivity for TUL is due to its strong lipophilic properties, which contrasts with inorganic cations that are highly hydrophilic, thus limiting their permeability and mobility through the hydrophobic membrane [56]. It is also evident that the increased hydrophobicity after adding MWCNT in sensor 2 contributed to greater selectivity values relative to the MWCNT-free ISM.

**Table 2:** The proposed sensor's logarithmic selectivity coefficients as determined by the separate solutions method (SSM)

Interferent (10^–4^ M)	${\text{logK }}_{\text{TUL}.\text{ I}}^{\text{pot}}$ Sensor 1	${\text{logK }}_{\text{TUL}.\text{ I}}^{\text{pot}}$ Sensor 2^a^
NaCl	− 2.99	− 3.14
KCl	− 3.00	− 3.34
MgCl_2_	− 3.33	− 3.83
CaCl_2_	− 3.19	− 3.68
Erythromycin	− 1.15	− 1.25
Clarithromycin	− 1.05	− 1.45
Ketoprofen	− 3.25	− 3.20

^a^average of three determinations

However, the selectivity towards TUL against structurally related molecules such as erythromycin and clarithromycin would be explained differently. Despite these drugs having comparable lipophilicity to TUL, the observed selectivity may be attributed to better binding affinity between the membrane/ionophore active sites and the TUL molecule. On the other hand, better selectivity values against ketoprofen, a commonly co-administered analgesic drug, can be linked to its acidic nature. The presence of an anionic center suggests incomplete ionization at the pH used, thereby reducing its ion exchange capacity within the membrane. The resulting selectivity offers an advantage when analyzing TUL in formulations and animal products containing both medications.

### Potential drift and water layer test

**Fig. 6:**
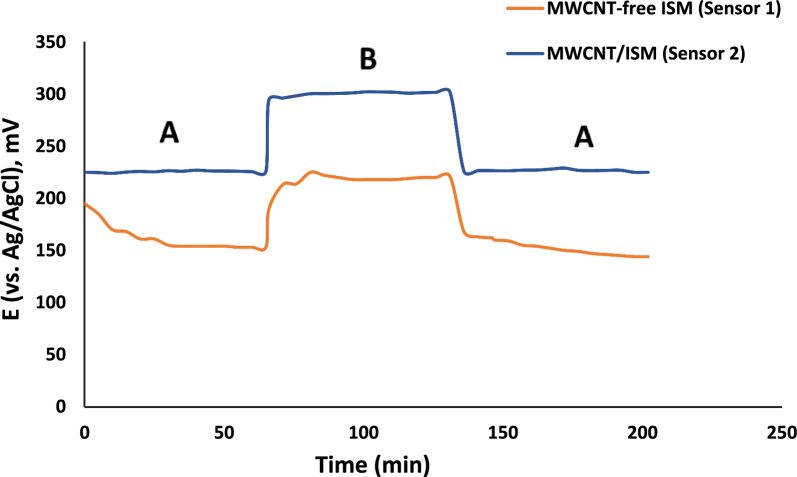
Water layer test of the two suggested sensors by recording potential against time in **A** 1.0 × 10^–3^ M TUL, **B** 1.0 × 10^–2^ M Diazepam HCl

**Fig. 7:**
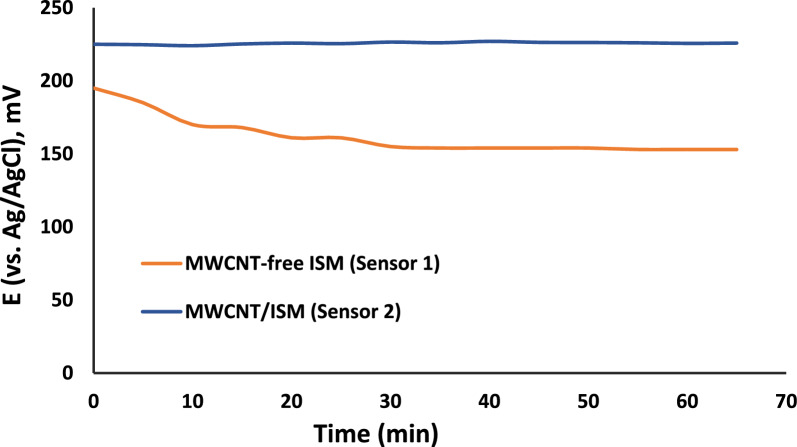
Study of potential drift over time for the two proposed sensors

The efficiency of SC-ISEs in real-time applications has been questioned due to potential drifts that compromise repeatability. The build-up of an aqueous layer between the solid substrate and the ISM has been deemed a major culprit for such variations in performance [57]. We conducted the water layer test to evaluate the existence of this disruptive film and the long-term stability of the optimized sensor. The test involved monitoring potential shifts in 1.0 × 10^–3^ M of TUL solution for one hour, followed by exposure to a highly concentrated solution of an interferent ion (Diazepam) with similar ionic behavior at the same pH for another hour before switching back to the primary ion solution. Figure 6 illustrates the superior stability of MWCNT/ISM, highlighting its enhanced resistance to the influx of interferent ions into any existing aqueous film. Furthermore, the introduction of MWCNT diminished the drift from 10.6 mV/h in sensor 1 to 0.6 mV/h in sensor 2, as evidenced by the potential stability observed within the first hour, Fig. 7. We can thus conclude that leveraging the lipophilic nature of MWCNT successfully hindered the production of an inner aqueous film, thereby mitigating instabilities and extending the sensor’s lifetime.

### Application in biological and non-biological samples

#### Potentiometric determination of TUL in Draxxin injection solution

The MWCNT/ISM sensor was successfully employed to assess TUL in its pharmaceutical formulation (Draxxin® solution) without prior sample extraction procedures. Results shown in Table 3 provided good percentage recovery (100.05 ± 1.28), suggesting that the developed sensor has the capability to accurately measure TUL in real samples without being affected by any additives present in the formulation.

**Table 3:** Quantification of TUL in Draxxin® injection using MWCNT/ISM sensor

Pharmaceutical formulation	Claimed concentration	Recovery ± RSD% ^a^
Draxxin® injection solution (Batch No.548396) labeled to contain (100 mg of TUL/mL)	4.96 × 10^–5^	99.60 ± 1.429
	4.96 × 10^–4^	100.51 ± 1.320
	Mean ± RSD%	100.05 ± 1.281

^a^average of three determinations

#### Potentiometric determination of TUL in Bovine liver and milk

The applicability of the MWCNT/ISE sensor was demonstrated in bovine food products. Table 4 presents the computed values for the quantity of TUL added, TUL found, and the recovery % for several spiked samples. Values of %R and %RSD fall within the acceptable analytical range, proving this technique valuable for direct and fast monitoring of TUL in milk and liver (within 8 ± 2s) without prior treatment. This finding is supported by the fact that the LOD value for sensor II (9.76 × 10^–8^ M) falls below the MRL of TUL at 4.5 ppm (5.58 µM) in the bovine liver [9]. As for milk, which is not permitted to include any TUL residues, the method can be utilized to identify potential instances of TUL misuse and administration during lactation periods.

**Table 4:** Determination of TUL in bovine products (bovine liver and milk) using MWCNT/ISM sensor

Bovine products	Spiked concentration (µM)	Found concentration (µM)^a^	Recovery (%)^a^	Mean recovery (%) ± RSD%
Liver	1 10 100	1.01 9.943 100.16	101% 99.43% 100.16%	100.19 ± 5.029
Milk	1 10 100	1.0184 9.742 101.14	101.84% 97.41% 101.14%	100.13 ± 6.121

^a^average of five determinations

#### Greenness assessment

In our commitment to conserving ecological resources and minimizing environmental impact, we assessed the proposed method’s greenness using the analytical Eco-Scale and Analytical GREEnness Calculator (AGREE).

The Semi-quantitative Eco-Scale approach involves giving penalty points to reagents and instrument parameters that deviate from the optimal green analysis practices in analytical methods [58]. Then, the sum of these points is deducted from 100 to give a final score that verifies the procedure as ideally green (> 75), satisfactory (> 50), or inadequate (< 50). As presented in Table 5, the proposed method scored 86, which is deemed excellent for a green method.

**Table 5:** Greenness evaluation of the suggested potentiometric approach utilizing Eco-Scale and AGREE tools

1) Analytical Eco-Scale	
Eco-scale hazard	Penalty points
**Reagents**	
Tetrahydrofuran	6
BRB buffer	2
**Instruments**	
Energy consumption	0
(< 0.1 kWh per sample)	
Occupational hazard	0
Waste	6
**Total penalty points (PP)**	14
**Eco-Scale total score**	86

2) AGREE	

Meanwhile, the AGREE metric system offers a comprehensive evaluation tool by considering each of the 12 principles of green analytical chemistry (GAC) [59]. Each principle is assessed on a scale from 0 to 1 in a clock-like diagram, with the total score in the center. As the score approaches 1, the corresponding segment gets greener. The resulting AGREE pictogram in Table 5, with a score of 0.83, underscores the high level of environmental compatibility achieved by the designed sensor.

#### Whiteness assessment

In contrast to the previous methods, the Whiteness approach not only focuses on the green component of an analytical method but also emphasizes the interdependence and harmony between analytical, environmental, and pragmatic facets [60]. This most recent approach applies the RGB12 algorithm to comprehensively evaluate the 12 principles of White analytical Chemistry (WAC) [61]. WAC utilizes a triadic color scheme to symbolize the key characteristics of the assessed methodology: red signifies analytical performance, green denotes ecological sustainability and safety, and blue represents practicality and affordability. The algorithm calculates scores for each color, yielding an overall score of “whiteness” that reflects the degree to which the method aligns with the WAC principles.

**Fig. 8:**
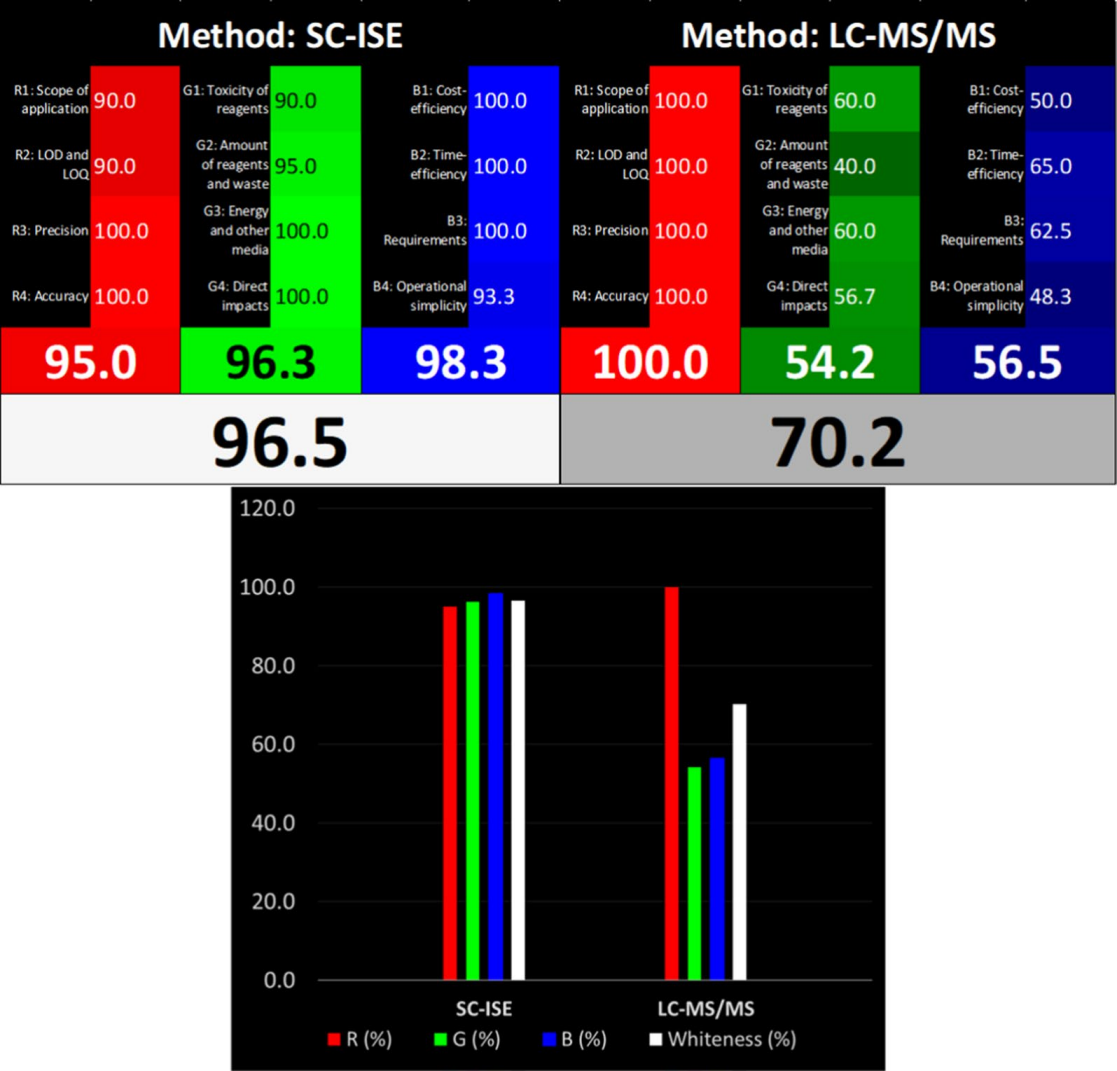
Comparison of the Whiteness of the proposed method (SC-ISE) against the reference method (LC–MS/MS) using the RGB12 model

We applied this approach to evaluate the overall sustainability of the proposed sensor in comparison to a reported chromatographic method [62]. As depicted in Fig. 8, our electro-sensing technique excels in green and blue areas, denoting superior functionality, simplicity, and environmental safety while performing sufficiently in the red area with sufficient accuracy and sensitivity. This achievement stems from employing less harmful reagents like BRB buffer instead of the non-green acetonitrile, minimizing energy consumption, and generating less waste through non-destructive measurements. Furthermore, the instrument is portable, far less expensive, significantly simpler, and requires much less time and effort than the HPLC–MS/MS instrumentation. Consequently, our proposed sensor achieves an impressive whiteness score of 96.5%, surpassing the HPLC–MS/MS score of 70.2%. We can conclude then that our proposed technique is a thoroughly balanced analytical method that adheres to sustainability standards and is specifically tailored to meet its objectives.

#### Merits of the novel sensor over conventional methods

To verify the validity of the designed sensor in determining TUL, statistical tests were used to compare the results with those of the HPLC method reported in the literature [20]. Table S-1 shows that the computed t- and F-values were lower than the tabulated ones, indicating no statistically significant difference between the accuracy and precision of the suggested and reported methods. Furthermore, a comparative study was conducted between the designated sensor and recently published work on TUL analysis. Table 6 highlights the originality of the newly designed sensor as the first potentiometric device capable of quantifying TUL in biological and non-biological samples. Our method offered precise detection at micromolar concentrations, surpassing the sensitivity of the HPLC/DAD by more than three orders of magnitude. The MWCNT/ISM sensor could detect TUL directly in as little as 8 s, whereas other methods required longer run times (around 10 min), not including the time needed for instrument preparation and arduous sample extraction (up to 80 min). Although these approaches could achieve lower detection limits, their high cost, potential hazards, and laborious nature render their applicability in real-time questionable. Alternatively, the proposed sensor is feasible, green, and suitable for in-situ analysis.

**Table 6:** A comparison between the developed and reported methods for TUL determination

Method	Application	Linearity range, detection limits	Operational simplicity and cost	Analysis time	Refs.
LC–MS/MS	Bovine muscle, fat, and liver	(0.025–100.0) ng/mL, 0.25 μg/kg	Lengthy sample preparation and extraction procedures (> 70 min), very expensive	7 min	[63]
LC–MS/MS	Urine, plasma, and seminal plasma in bull	(0.1–10), (0.05–5.0), (0.01–1.0) μg/mL for each matrix, respectively, no specified LOD	Lengthy sample preparation and extraction procedures (> 80 min), very expensive	> 5 min	[12]
HPLC–DAD	Injection solution	(1000.0—3000.0), 184.43 µg/mL	No extraction procedures, relatively expensive	10 min	[20]
Voltammetric sensor	Swine liver, flesh, sebum	(0.006–2.42) µg/mL, 0.001 ng/mL	Requires extraction procedures (> 30 min)	NA	[25]
Potentiometric sensor	Dosage form Bovine liver and milk	(0.08–806.1), 0.078 µg/mL	No need for extraction procedures, portable and cost-effective	8 s	This work

## Conclusion

The present work introduces a novel potentiometric sensor for quantifying TUL in various biological and non-biological matrices. The designed sensor displayed promising precision, accuracy, speed, and durability. Integrating carbon nanotubes effectively controlled the water layer production and stabilized the membrane response. Beyond that, the MWCNT/ISM sensor exhibited superior performance, featuring a lower limit of detection, faster response time, greater selectivity, minor drift, and longer shelf-life than the MWCNT-free sensor. The modified sensor could accurately quantify TUL in its dosage form with minimum interference from coexisting ions. This capability extended to the analysis of milk and liver samples, obviating the need for complex extraction procedures required by previous methods. Based on the greenness and whiteness profiles analysis, our method stands out for its convenience, sustainability, and affordability, proving it a reliable option for monitoring TUL levels in various matrices.

### Supplementary Information

Supplementary file 1.

## Data Availability

The datasets used and/or analyzed during the current study are available from the corresponding author upon reasonable request.
