# Supplementary material for: Eco-conscious potentiometric sensing: a multiwalled carbon nanotube-based platform for tulathromycin monitoring in livestock products
Source: BMC Chem. 2024 Aug 12;18(1):151. doi: 10.1186/s13065-024-01255-7 (PMC11318228; doi:10.1186/s13065-024-01255-7)
Supplement: Supplementary file 1 [file 13065_2024_1255_MOESM1_ESM.docx]

# Supplementary Information

# For

**Eco-conscious potentiometric sensing: a multiwalled carbon nanotube-based platform for tulathromycin monitoring in livestock products**

Omnia G. Hussein ^a,*^, [Hany H. Monir](https://www.tandfonline.com/author/Monir%2C+Hany+H) ^a^ , Hala E. Zaazaa ^a^, Maha M. Galal ^a^

^a^ Analytical Chemistry Department, Faculty of Pharmacy- Cairo University, Kasr El Aini Street, Cairo 11562, Egypt

^*^ Corresponding author, [omneia.gamal@pharma.cu.edu.eg](mailto:omneia.gamal@pharma.cu.edu.eg) (O.G. Hussein), +201030154959

**Sensing mechanism of TUL sensor**

Potentiometry relies on measuring the potential difference between two electrodes in an electrochemical cell to determine an analyte ion of interest, TUL in this case. The sensing device consists of a reference Ag/AgCl electrode and an ion-selective electrode for TUL. The sensor potential (emf) is measured relative to the reference electrode, which provides a constant sample-independent half-cell potential under zero-current conditions [1]. This electrical potential—known as the phase-boundary potential—develops at the interface of the ISM and the sample solution due to the selective distribution of TUL ions between these two phases, leading to the interfacial separation of charges [2,3]. The output potential is directly proportional to the concentration of the targeted ion in the solution, as described by the Nernst equation:

*𝑒𝑚𝑓 = 𝐸^0^ + (R𝑇/𝑧F) ln 𝑎*

R, T, and F denote the universal gas constant, temperature, and Faraday constant. z and a are the target ion's charge and activity. E^0^ denotes the total of other boundary potentials that must remain constant. Accordingly, a ten-fold change in TUL activity should lead to a predicted 19.7 mV change.

The ISM was designed to select TUL ions based on TUL’s lipophilic nature and the host-guest interaction with a CX-6 ionophore. The cationic selectivity for TUL’s positively charged ions was imparted by doping the membrane with hydrophobic anionic sites using tetrakis 4-chlorophenyl borate salt [4]. Because the concentration of TUL in the ISM is fixed by the anionic sites’ concentration, the potential at the phase boundary potential is solely dependent on the activity of TUL in the sample solution within the linear range of the electrode [2, 5]. Therefore, any alteration in the concentration of TUL ions will promptly reestablish equilibrium and modify the potential at the phase boundary. The solid contact on the surface of GCE, enhanced by the inclusion of carbon nanotubes, is responsible for transducing the ionic signal into a quantifiable electrical signal [6].


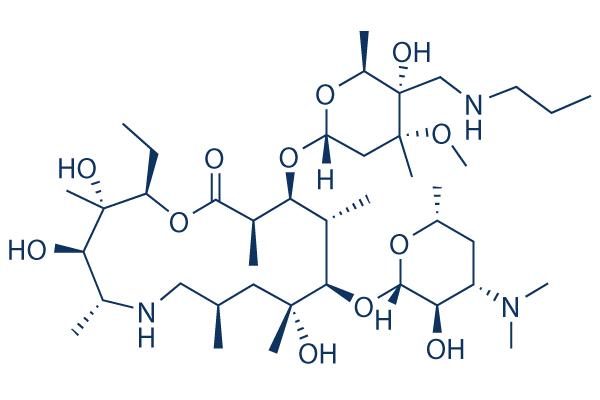


# Fig. S-1. Chemical structure of Tulathromycin

Fig. S-2. Effect of different plasticizers on the potentiometric response of TUL sensing membranes.


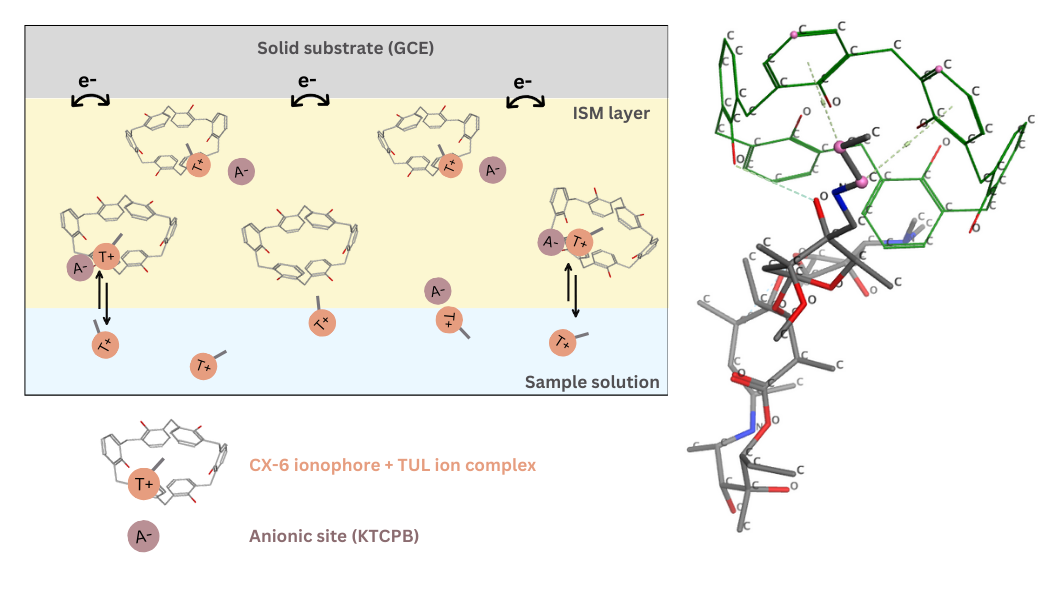


Fig. S-3. A schematic showing the interaction between TUL ions and the active ionophore sites in the ISM.

Fig. S-4. The calibration curves of membranes with different MWCNT ratios


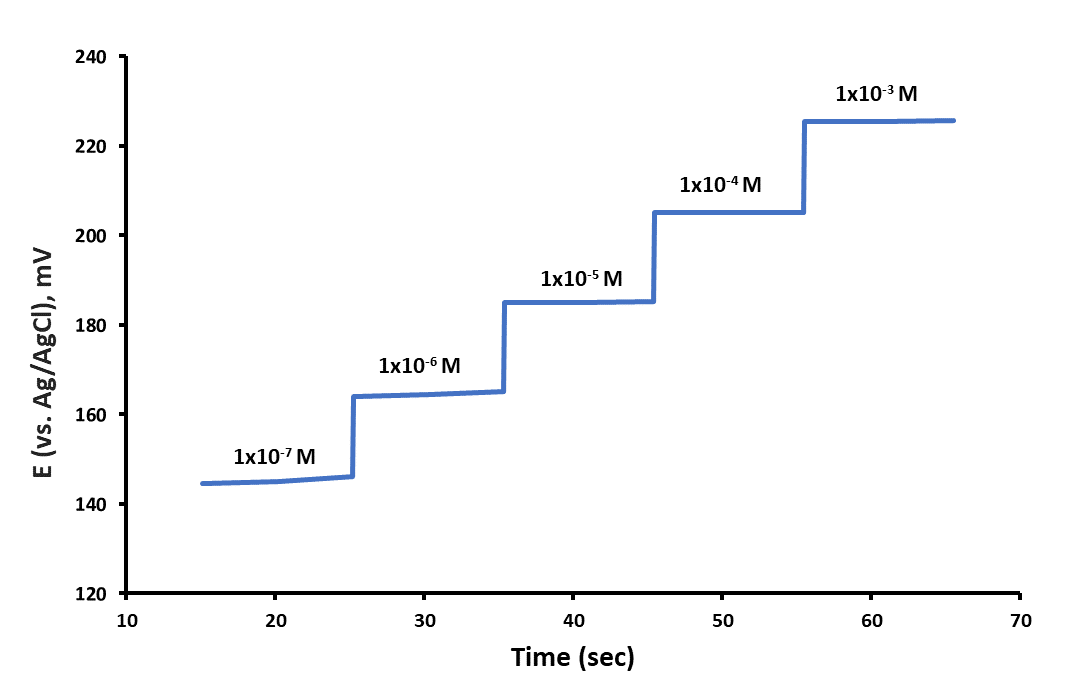


Fig. S-5. Dynamic response time of MWCNT/ISE sensor towards increasing concentrations of TUL solutions.

**Table S-1:** Statistical comparison of MWCNT/ISM sensor results with the HPLC reported method for pure powdered TUL determination.

| Item | MWCNT/ISM sensor | Reported Method ^a^ |
| --- | --- | --- |
| Mean ± S.D. | 99.25 ± 0.657 | 98.95 ± 0.670 |
| Variance | 0.432 | 0.449 |
| n | 5 | 5 |
| Student’s t-test^b^ | 0.715 (2.776) |  |
| F-value^b^ | 1.040 (6.39) |  |

^a^ HPLC method using a C18 column with a mobile phase consisting of a mixture of (methanol: Phosphate buffer) (85:15), flow rate 1.0 mL/min, and detection wavelength at 210 nm.
^b^ The values in parentheses are the corresponding theoretical values for t and F at P = 0.05.

**References:**

1. Bühlmann P, Chen LD. Ion‐Selective Electrodes With Ionophore‐Doped Sensing Membranes. In: Supramolecular Chemistry. Wiley; 2012.

2. Bakker E, Nägele M, Schaller U, Pretsch E. Applicability of the phase boundary potential model to the mechanistic understanding of solvent polymeric membrane‐based ion‐selective electrodes. Electroanalysis. 1995;7:817–22.

3. Bakker E, Bühlmann P, Pretsch E. Carrier-Based Ion-Selective Electrodes and Bulk Optodes. 1. General Characteristics. Chem Rev. 1997;97:3083–132.

4. Amemiya S. Potentiometric Ion-Selective Electrodes. In: Handbook of Electrochemistry. Elsevier; 2007. p. 261–94.

5. Mousavi MPS, Abd El-Rahman MK, Mahmoud AM, Abdelsalam RM, Bühlmann P. In Situ Sensing of the Neurotransmitter Acetylcholine in a Dynamic Range of 1 nM to 1 mM. ACS Sens. 2018;3:2581–9.

6. Shao Y, Ying Y, Ping J. Recent advances in solid-contact ion-selective electrodes: functional materials, transduction mechanisms, and development trends. Chem Soc Rev. 2020;49:4405–65.
